# Lipid accumulation patterns and role of different fatty acid types towards mitigating salinity fluctuations in *Chlorella vulgaris*

**DOI:** 10.1038/s41598-020-79950-3

**Published:** 2021-01-11

**Authors:** Kit Yinn Teh, Saw Hong Loh, Ahmad Aziz, Kazutaka Takahashi, Abd Wahid Mohd Effendy, Thye San Cha

**Affiliations:** 1 Faculty of Science and Marine Environment, Universiti Malaysia Terengganu, 21030 Kuala Terengganu, Terengganu Malaysia; 2 Satreps-Cosmos Laboratory, Central Laboratory Complex, Universiti Malaysia Terengganu, 21030 Kuala Terengganu, Terengganu Malaysia; 3 Institute of Marine Biotechnology, Universiti Malaysia Terengganu, 21030 Kuala Terengganu, Terengganu Malaysia; 4 Department of Aquatic Bioscience, Graduate School of Agricultural and Life Sciences, The University of Tokyo, 1-1-1, Yayoi, Bunkyo-ku, Tokyo, 113-8657 Japan

**Keywords:** Lipids, Fatty acids, Oils, Biofuels, Biodiesel, Salt

## Abstract

Mangrove-dwelling microalgae are well adapted to frequent encounters of salinity fluctuations across their various growth phases but are lesser studied. The current study explored the adaptive changes (in terms of biomass, oil content and fatty acid composition) of mangrove-isolated *C. vulgaris* UMT-M1 cultured under different salinity levels (5, 10, 15, 20, 30 ppt). The highest total oil content was recorded in cultures at 15 ppt salinity (63.5% of dry weight) with uncompromised biomass productivity, thus highlighting the ‘trigger-threshold’ for oil accumulation in *C. vulgaris* UMT-M1. Subsequently, *C. vulgaris* UMT-M1 was further assessed across different growth phases under 15 ppt. The various short, medium and long-chain fatty acids (particularly C20:0), coupled with a high level of C18:3n3 PUFA reported at early exponential phase represents their physiological importance during rapid cell growth. Accumulation of C18:1 and C18:2 at stationary growth phase across all salinities was seen as cells accumulating substrate for C18:3n3 should the cells anticipate a move from stationary phase into new growth phase. This study sheds some light on the possibility of ‘triggered’ oil accumulation with uninterrupted growth and the participation of various fatty acid types upon salinity mitigation in a mangrove-dwelling microalgae.

## Introduction

Lipid metabolites play an important role in oleaginous microalgae because the fatty acids that are incorporated into various lipid groups are a major component of the microalgae cell and essentially involved in biochemical processes, building of membrane structures, signalling and storage^1,2^. Of the various lipid metabolites, storage lipids, mainly triacylglyceride (TAG), remains an interesting target as its rapid increase can be easily manipulated by culture duration and culture medium manipulation^3^. Microalgae accumulate TAG readily as the component is easy to breakdown thus making it easier for the cell to overcome adverse environmental conditions. Fatty acids that get functionalized into storage lipids are concomitantly produced with the induction of other lipid biosynthetic pathways^4^, thus the resultant fatty acid composition is an extension of its metabolic reactions to environmental stimuli and stress. Due to the diverse phylogeny of microalgae, significant differences do occur in the types of fatty acids partitioned into TAG upon reception of salinity stress. For example, it was observed that Chlorophyta tended to synthesises saturated fatty acids (SFA) and monounsaturated fatty acids (MUFA) while Ochrophyta (diatoms) preferred production of polyunsaturated fatty acids (PUFA) when stressed^5^. However, regardless of phylogeny, oleaginous microalgae will rearrange its lipid pathways in order to accumulate high amounts of TAG (about 40–70% per dry weight) upon exposure to abiotic stress conditions^4,6^.

Of the various abiotic stresses, the application of salinity stress-mediation to increase TAG yield has been consistently and extensively studied on both marine and freshwater microalgae and occasionally, on euryhaline microalgae. In most studies, salinity stress was observed to boost yield of TAG most effectively under conditions that challenge microalgae biomass growth as salinity levels may exceed the limits of salinity tolerance of the microalgae in study. Due to these circumstances, studies that use salinity stress to boost TAG yield often involve halotolerant or halophilic microalgae capable of tolerating hypersaline conditions^7–9^. Collectively, these types of experiments imply that salinity stress can only be applied to certain types of microalgae. By doing so, we are limiting source diversity as well as the potential of information and resource we may be able to tap from various microalgae. Expending bioprospecting for more efficient TAG-accumulating microalgae species is more beneficial and ever more so important during current times of climate change^10^.

Mangroves are interesting ecosystems with daily transition gradients between freshwater and marine environments. For this reason, mangrove-dwelling microalgae strains may carry inherent adaptative strategies that may hold the key to overcoming the ‘high yield—low biomass’ conundrum but are unfortunately lesser studied^11^. Hence, experiments carried out at very high salinities (up to 150 parts per thousand (ppt)) may not be able to properly represent physiological aspects of mangrove-dwelling microalgae. Therefore, this current study first seeks to understand how mangrove-isolated *Chlorella vulgaris* UMT-M1 responded to different salinity levels in terms of biomass, oil and fatty acid production. After the salinity level coinciding with highest oil accumulation had been established, which in this case was 15 ppt, the study sought to understand the adaptative response (in terms of fatty acid production), which extended from the early exponential to late stationary growth phase. It was interesting to observe how the cell mitigated salinity fluctuation via the changes in fatty acid types partitioned into TAG during the different growth phases. While the roles of the different fatty acids remained unclear, this study provided fundamental comparison on the fatty acid composition alteration between points of contact with salinity change. The findings also show that mangrove-isolated *C. vulgaris* UMT-M1 may provide new perspectives towards deciphering the high yield—low biomass conundrum often encountered in stress-mediated strategies.

## Results

### Growth curve under different salinity measurements

**Figure 1:**
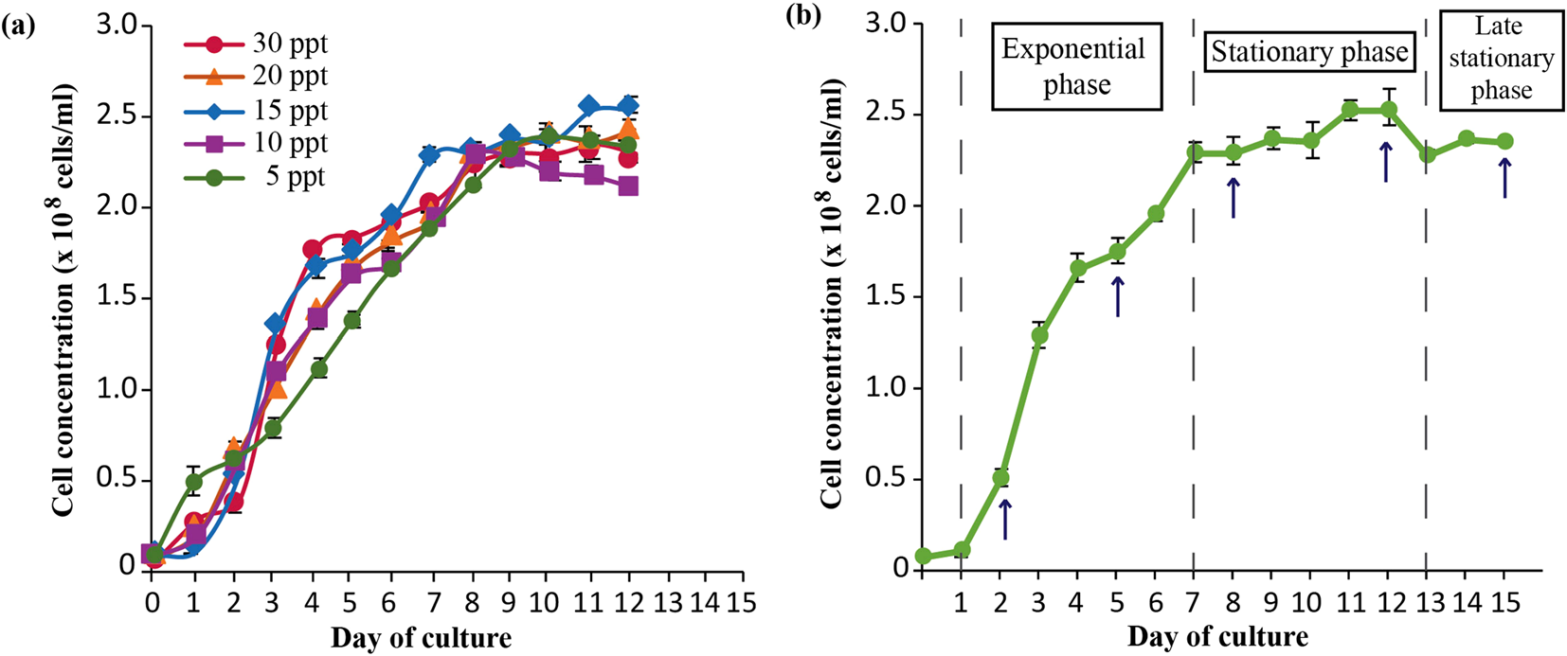
Growth curve of *C. vulgaris* UMT-M1 **(a)** cultivated under different salinity measurements of 5, 10, 15, 20 and 30 ppt (with 30 ppt as control). The cultures were harvested at stationary phase at day 12 for further analysis. **(b)** Cultivated under the selected salinity of 15 ppt. Cultures were harvested at early exponential phase (day 2), mid-exponential phase (day 5), early stationary phase (day 8), mid-stationary phase (day 12) and late stationary phase (day 15). All values presented represent the mean ± SD (n = 3). Arrows (↑) indicate the day at which the cells from three random replicates were harvested for analysis.

In the control (30 ppt) and all screened salinities (5, 10, 15, 20 ppt), UMT-M1 possessed a growth curve that exhibits three separate growth phases; i.e. lag phase (day 0–2), exponential phase (day 3–7), and stationary phase (day 8–12) (Fig. 1a). Cultures in all salinities entered stationary phase at about the same point which was at day 8 (Fig. 1a). The cultures were harvested at day 12 for further assessments. At day 12, cultures in 15 and 20 ppt achieved higher (*p* < 0.05) cell concentrations of between (2.5–2.6) × 10^8^ cells mL^−1^ compared to 2.3 × 10^8^ in the control culture (30 ppt), while the lowest cell concentration was recorded in 10 ppt cultures with 2.1 × 10^8^ cells mL^−1^ (Table S1). Unexpectedly, cell concentration in the lowest salinity (5 ppt) appeared to be the intermediate with cultures achieving cell concentrations comparable to the 30 ppt (control) and 20 ppt which ranged between (2.3–2.4) × 10^8^ cells mL^−1^ (Fig. 1a; Table S1).

### Effects of salinity level on growth parameters, chlorophyll and total oil content under different salinity measurements

**Figure 2:**
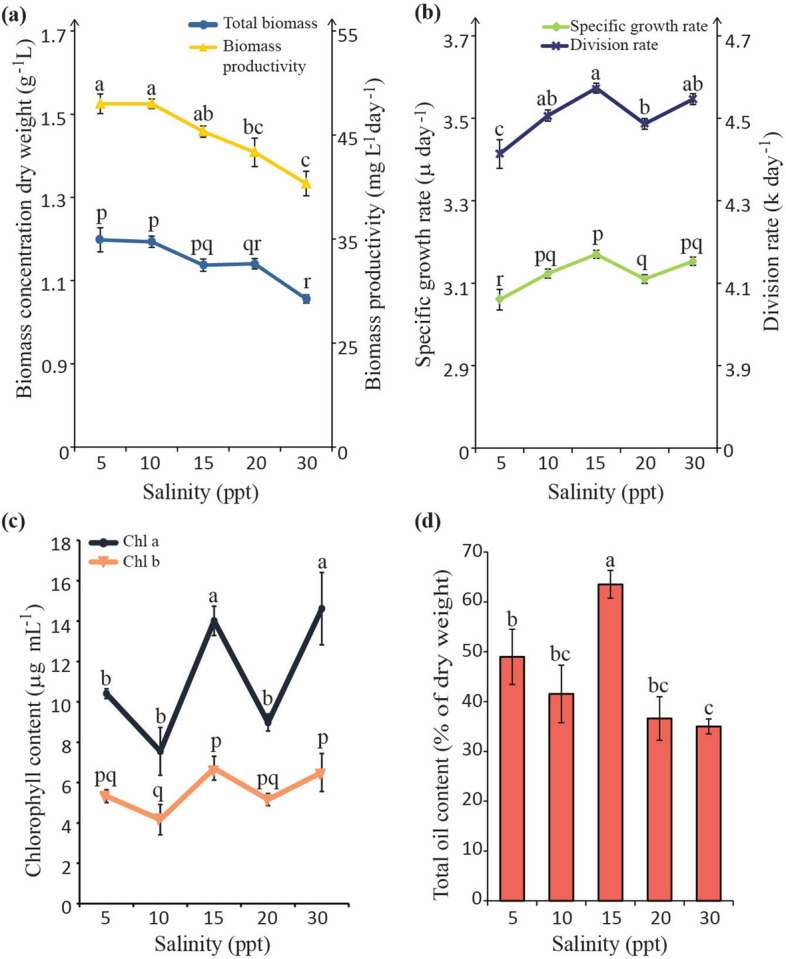
Effect of different salinities on **(a)** biomass production and biomass productivity; **(b)** specific growth rate (µ) and division rate (*k*); **(c)** chlorophyll content and **(d)** total oil content of *C. vulgaris* UMT-M1. Data presented are means ± SD of three biological replicates. Different series of letters represent significant differences within each parameter measured (Fisher’s LSD (SPSS software), *p* < 0.05).

On average, total dry biomass accumulated in the five different salinities ranged between 1.06 and 1.2 g L^−1^ with biomass production increasing as salinities decrease (Fig. 2a). Biomass accumulated in the control was significantly lower in comparison to 5, 10 and 15 ppt cultures which accumulated between1.14 to 1.20 g L^−1^, of dry biomass (Fig. 2a). Total dry biomass recorded in 20 ppt (1.08 g L^−1^) was however not significantly different from the control (30 ppt). Although final cell density data showed a more dispersive pattern (Fig. 1a; Table S1), there exists an inversely proportional relationship between the factors of salinity and total biomass accumulation as this pattern was reflected in their biomass productivities as well (Fig. 2a). Higher biomass productivities, in comparison with the control, were recorded at 5, 10 (48 mg^−1^ day^−1^) and 15 ppt (45 mg^−1^ day^−1^) but there was no significant difference between 20 ppt cultures (43 mg^−1^ day^−1^) and the control (40 mg^−1^ day^−1^) (Fig. 2a). Specific growth rate (*µ*) and division rate per day (*k*) on the other hand, measures how growth proceeds during the exponential phase of growth. Differing from the patterns seen in total biomass and productivities, the graphs do not exhibit the gradual linear arrangement (Fig. 2b). It instead shows fluctuating patterns with rate measurements in 10 ppt (*µ*, 3.12; *k*, 4.51) and 20 ppt (*µ*, 3.11; *k*, 4.49) cultures sitting in between salinities possessing the significantly higher (15 ppt: *µ*, 3.17; *k*, 4.57 and 30 ppt: 3.15; *k*, 4.55) and lowest (5 ppt: *µ*, 3.06; *k*, 4.41) rates (Fig. 2b).

In the case of chlorophyll content, the two major pigments in green microalgae; i.e. chl a and chl b were measured. Both chl a and chl b contents fluctuated across the salinity treatments (Fig. 2c). Notably, chl a (14.01 µg mL^−1^) and chl b (6.71 µg mL^−1^) content in 15 ppt were comparable to the control (chl a: 14.62 µg mL^−1^; chl b: 6.5 µg mL^−1^). However, chl a content in the remaining salinities was significantly lower than the control, ranging between 7.6 and 10.4 µg mL^−1^ (Fig. 2c). In contrast, chl b content showed an insignificant fluctuating pattern across the tested salinities, with the lowest value being recorded in 10 ppt (4.16 µg mL^−1^) while values in 5 ppt (5.33 µg mL^−1^) and 20 ppt (5.16 µg mL^−1^) were comparatively lower against the control (Fig. 2c).

Concurrently, the highest total oil content was achieved under 15 ppt conditions, recording a value of 63.5% (percentage of dry weight basis). This is an increase of 1.8 fold from the oil content recorded in control conditions (35.0%). This was followed by a 1.3-fold increase as seen in 5 ppt conditions, which recorded total oil content of 49% compared to control (Fig. 2d).

### Response of fatty acid profile in different salinity measurements

**Figure 3:**
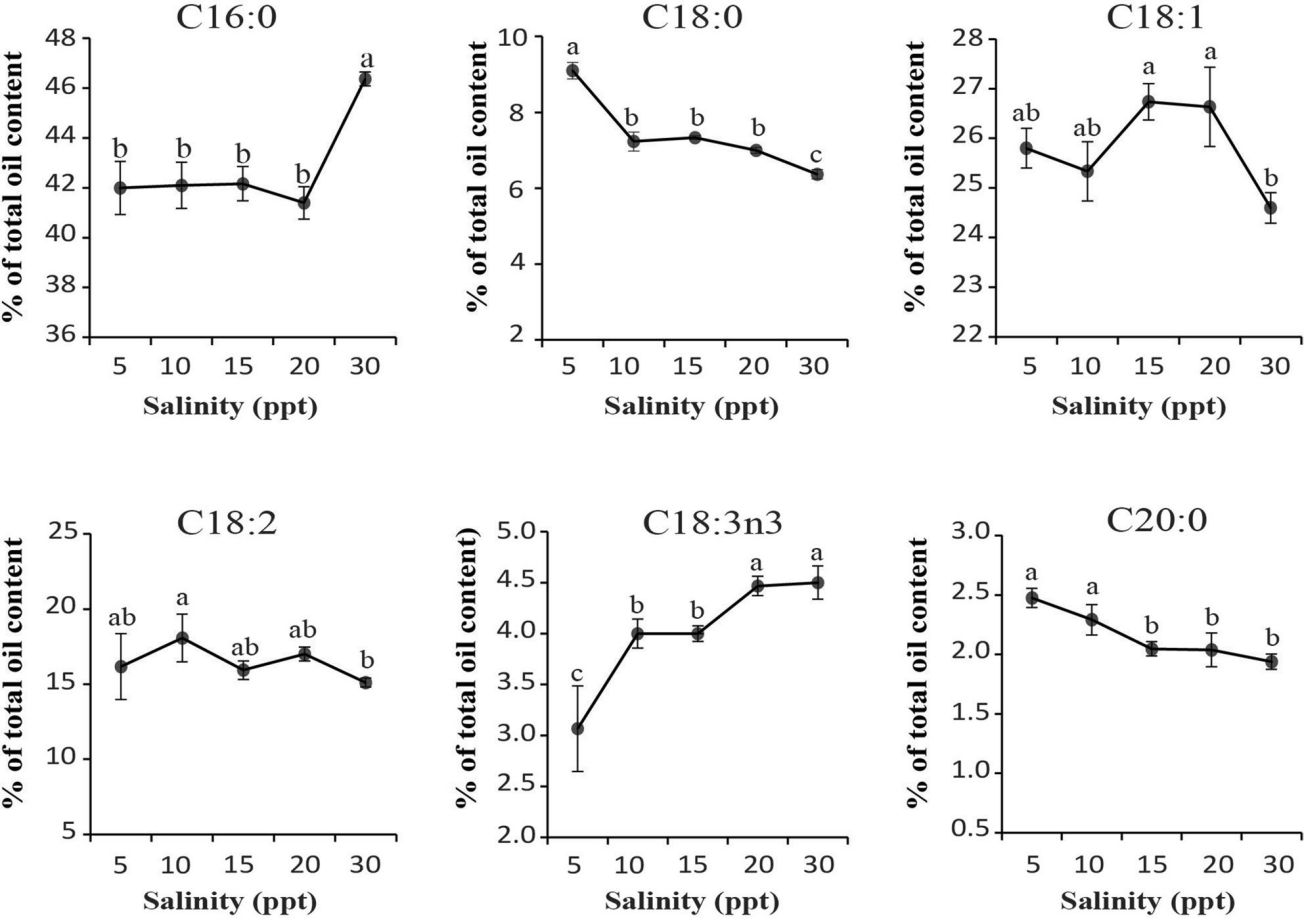
Distribution of changes in composition of the major fatty acids under different salinity treatments in *C. vulgaris* UMT-M1. All values represent means ± SD of three biological replicates. Different series of letters are significantly different according to Fisher’s LSD test (SPSS software) (*p* < 0.05).

The total SFAs, MUFAs and PUFAs were consistent across all salinity measurements. The most dominant fatty acid in *C.vulgaris* UMT-M1 was palmitic acid (C16:0) which range from 42 to 46.4%, followed by oleic acid (C18:1) with 24.6–26.6%, with lesser amounts of PUFA such as linoleic acid (C18:2) and α-linolenic acid (C18:3n3). Combined, they make up > 96% of all the detectable fatty acid types. The remaining percentage are accounted for by fatty acid types that measure below 1% and are considered negligible and would not be discussed in this report. Both, C16:0 and C18:0 show diverging patterns in the lowest and highest salinities (Fig. 3). The production of C16:0, was significantly highest in the control (30 ppt) with 46.37%, while C18:0 production peaked in 5 ppt cultures with 9.1% (Fig. 3). Production of C16:0 in the other salinities (5–20 ppt) averaged around 41.4–42.1% (*p* > 0.05). Functionally, being a metabolite in the concomitant step towards fatty acid elongation and desaturation, C18:0 levels were the lowest (*p* < 0.05) in control (6.37%) and remained significantly lowest than 5, 10, 15 and 20 ppt cultures (Fig. 3). In the case of C18:1 and C18:2, both fatty acids recorded fluctuating amounts (between 25.3–26.6% and 15.9–18.1% respectively) across the different salinities. The PUFA C18:3n3 showed rather interesting patterns with a stepwise decline from 4.4 to 4.7% in the high salinities (20 and 30 ppt) to the lowest level of 3.2% in 5 ppt (Fig. 3). The C20:0 and a cumulative of some minor fatty acids (FAs) are found making up a small portion of the detectable fatty acids. The amounts of C20:0 was observed as significantly increased towards the lower salinities of (5 and 10 ppt) (Fig. 3). Overall, different salinity measurements do not appear to drastically alter the fatty acid composition of *C. vulgaris* UMT-M1. Conjunctively, the PCA score plot of the fatty acid profile showed distinct clustering among replicates of the control (30 ppt), 20 and 15 ppt but looser clustering patterns in cultures of the lower salinities of 5 and 10 ppt (predicted accounted variance up to 89.4% (R^2^X = 0.894)) (Supplementary Fig. S1). PCA analysis revealed that there were no outliers detected in the fatty acid compositions from the tested salinity concentrations (Supplementary Fig. S1).

### Performance of *C. vulgaris* UMT-M1 at different growth stages under 15 ppt salinity measurement

On the basis of highest total oil production (63.5%), along with stable chlorophyll content under 15 ppt, this salinity was further assessed along its growth phases. Figure 1b shows the growth curve of *C. vulgaris* UMT-M1 cultivated under 15 ppt salinity for 15 days. The phases studied were at early exponential phase (day 2), mid-exponential phase (day 5), early stationary phase (day 8), mid-stationary phase (day 12) and the late stationary phase (day 15) (Fig. 1b).

### Biomass production and total oil content under 15 ppt salinity measurement

**Figure 4:**
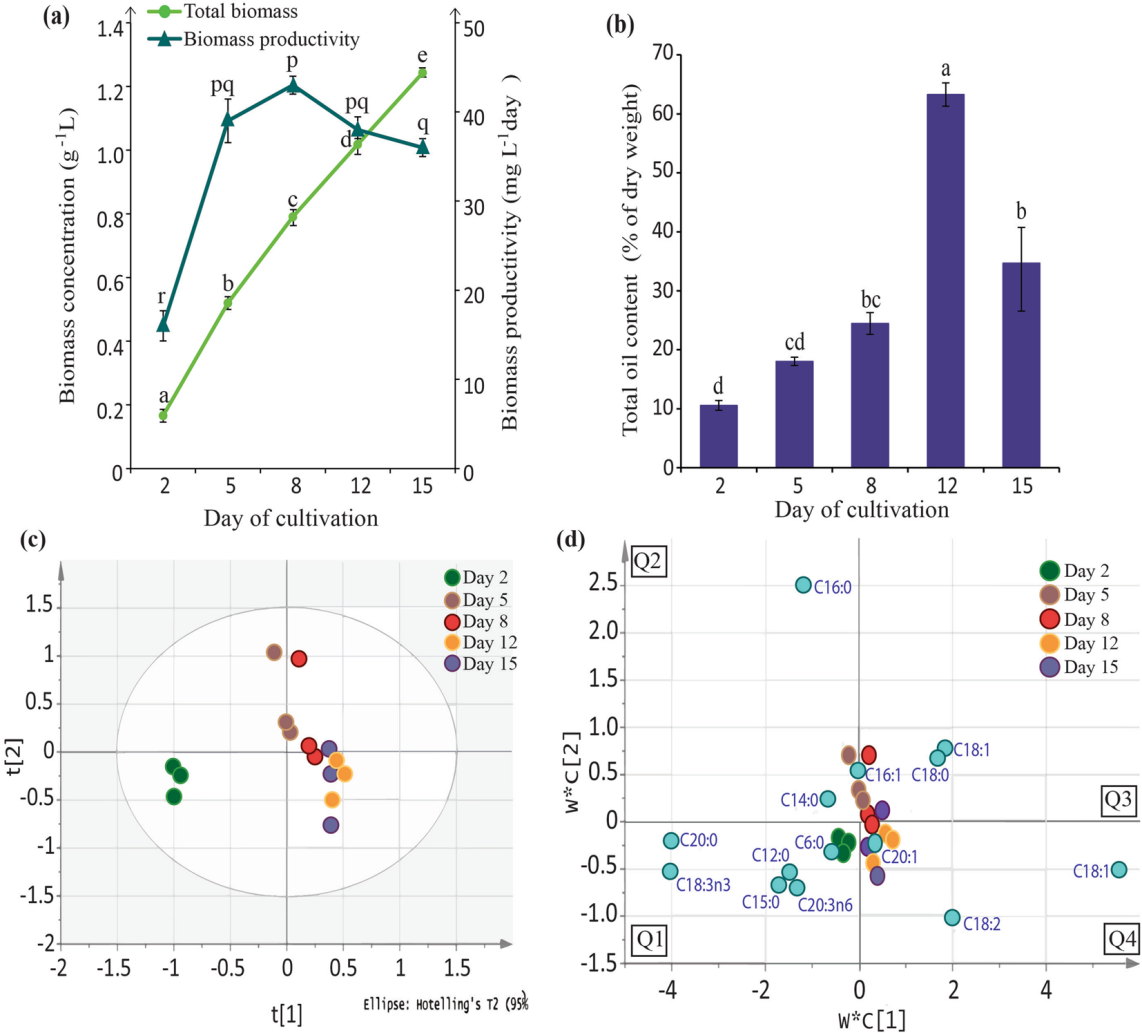
Changes in **(a)** biomass production and biomass productivity, **(b)** total oil content along different growth phases observed at day 2, 5, 8, 12 and 15. *C. vulgaris* UMT-M1 cultures grown under selected salinity of 15 ppt. All values represent the mean ± SD from three biological replicates. Different series of letters represent significant differences within each parameter measured (Fisher’s LSD (SPSS software), *p* < 0.05). Prediction of fatty acid profile was carried out using PLS-DA (SIMCA) where (**c**) score plot shows how closely related each time-points are while (**d**) generated PLS-DA scores loadings plot show the predominant fatty acid changes through the growth phases. Loadings (blue dots) represent fatty acid type. Eclipse in **(c)** represents 95% confidence level. (R^2^X = 0.714, R^2^Y = 0.97, Q^2^ = 0.984). Q1–Q4 represent the 4 quadrants of the score/loadings plot.

Total biomass was lowest at day 2 (0.17 g L^−1^) and increased gradually during day 5 (0.52 g L^−1^), day 8 (0.79 g L^−1^), day 12 (1.02 g L^−1^) and eventually achieving its highest dry weight at day 15 (1.24 g L^−1^) (Fig. 4a). Biomass productivity however paints a different picture (Fig. 4a). Biomass productivity produces a sharp incline pattern during day 2–day 8 (20–44 mg L^−1^ day^−1^), coinciding with exponential growth in which cells are actively dividing. A decline pattern from day 8–day 15 (44.0–35.0 mg L^−1^ day^−1^) was observed, in line with the stationary phase where cell division ceases. This was in contrary to biomass production which incurred its highest reading at day 15 (Fig. 4a).

Total oil content gradually increased from the lowest at day 2 (10.6%), day 5 (18.0%), day 8 (24.5%) until eventually achieving the highest total oil content at day 12 (63.3%). Total oil content however dropped after day 12. At day 15, total oil content recorded only half (34.7%) of the fraction achieved at day 12 (Fig. 4b).

### Breakdown of fatty acid profile changes at different growth phases

Data about sample variation was deduced using partial least squares discriminant analysis (PLS-DA) which showed overall model of the patterns of changes occurring in the fatty acid profile from day 2, 5, 8, 12 and 15 (Fig. 4c). It can be observed that day 2 (green) clustered the farthest away from the other time-points while clusters of days 5,8 (pink and red) and days 12,16 (yellow and purple) clustered side by side respectively (Fig. 4c). This may point to strongly different fatty acid profiles between the different days of cultures. No outliers were detected. Scores loadings plot (Fig. 4d) of the PLS-DA analysis is used to deduce the factor (in this case fatty acid type) responsible for the cluster pattern observed in Fig. 4c. As depicted in Fig. 4d, clusters of day 2 (in Q1) were affected by the presence of more fatty acid types, particularly short-chained fatty acids (SCFA)such as C6:0, C12:0 and C15:0 along with two types of PUFA (C18:3n3, 20:3n6). In the quadrants Q2 and Q3, clusters of day 5 and 8 appear to be marked by a variety of SFAs (C14:0, C16:0, C16:1, C18:0) and MUFA C18:1. In quadrant Q4, the fatty acids affecting clustering of day 12 and 15 are C18:2, C18:1 and C20:1. The PLS-DA model generated possesses high degree of fit to data (R^2^X = 0.714), high explained variation (R^2^Y = 0.97) and high measures of consistency (Q^2^ = 0.984). Though the model only predicts the strongest variables (fatty acid type) affecting each day, a more detailed day-to-day layout of the fatty acid profiles can be observed in Supplementary Fig. S2.

**Figure 5:**
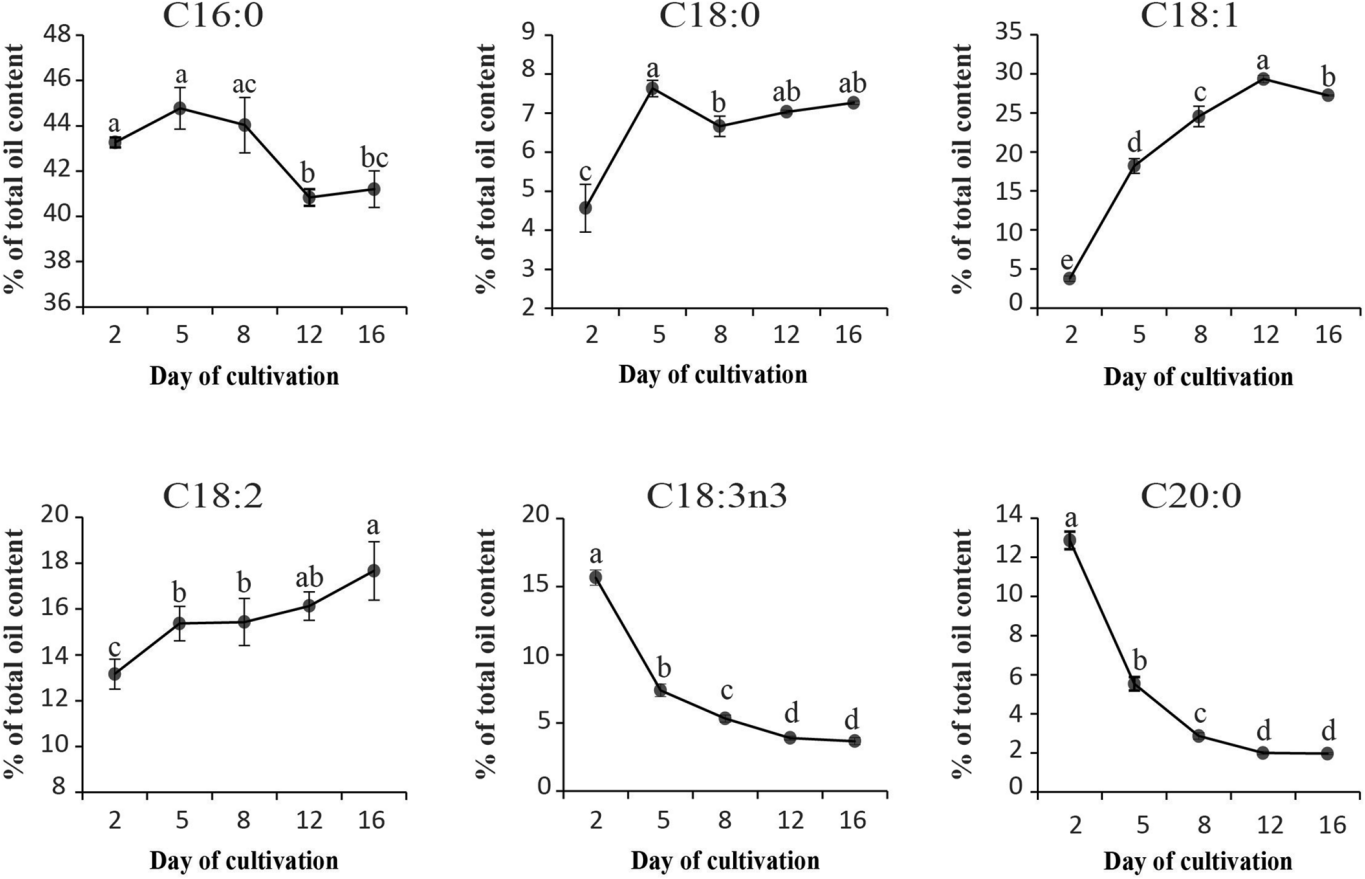
The major fatty acid compositions of *C. vulgaris* UMT-M1 at different growth phases under 15 ppt culture condition. All values represent means ± SD of three biological replicates. Different series of letters are significantly different according to Fisher’s LSD test (SPSS software) (*p* < 0.05).

Both SFA and PUFA showed a depreciating trend against an appreciating trend for MUFA. The total SFA (mainly contributed by C16:0) demonstrated a gradual decline from 65.7% at early exponential phase (day 2) to between 50.2 and 49.9% at day 12 and day 15. Conversely the total MUFA (predominantly C18:1) increased drastically from a mere 4.13% at day 2 to 29.7% at stationary phase (day 12) (Supplementary Fig. S3).The SFAs, C16:0 and 18:0 depicted opposing accumulation patterns towards later stationary phases (Fig. 5). While amounts of C16:0 was generally maintained (*p* > 0.05) from day 2–8 (43.4–44%), it experienced a significant decrease as cultures entered the later stationary phases at day 12–15 (41.2–40.8%). Contrastingly, C18:0 levels were the lowest at day 2, recording only 4.6% before an increase to 7.6% by day 5 and maintained thereafter from days 8–16 (6.7–7.3%) (Fig. 5). On the other hand, C18:1 staged a significant increase in amount across each growth phase from the lowest at day 2 (3.8%) to the highest at day 12 (29.3%) before experiencing a significant decrease at the late stationary phase which recorded 27.2% (Fig. 5). Comparing both PUFA types, although C18:2 showed a gradual increase ranging between 13.2 and 17.7% as the cultures progressed from the early exponential phase (day 2) into the late stationary phase (day 15), the C18:3n3 experienced a sharp decline during the period (Fig. 5). The C18:3n3 recorded the highest amount of 15.7% at day 2, followed by a steady decline (*p* < 0.05) to only 3.7% at day 15. Most interestingly, this pattern was seen to coincide with the accumulation of several other short and medium chain saturated fatty acid (SC/MC-SFA) types such as C6:0, C12:0, C14:0 and C15:0 as well as long chained SFAs (LC-SFA) such as C20:0. Among these, the C20:0 content which accounts for 12.9% was substantially highest at day 2, coinciding with active cell division, followed by steep decline to about 2% towards mid and late stationary phase (Fig. 5).

## Discussion

### Growth performance of *C. vulgaris* UMT-M1 at different salinity cultivation conditions

Preference for a particular salinity range was not detected as all cultures attained similar growth phases with the control (Fig. 1a, Table S1). Although biomass yield of freshwater strains of *C. vulgaris* improved under low levels of salinity^12^, growth was mostly reported to be hindered in concentrations leading up to full strength seawater^13^. Biomass accumulation in *C. vulgaris* UMT-M1 however, was not drastically reduced along the tested salinity gradient, affirming its tolerance for a wide range of salinity levels (Fig. 2a). In fact, most euryhaline microalgae are known to commonly display non-selective growth patterns towards a wide range of salinities^10,14^. Incidentally, it had been observed that nitrate assimilation from culture medium may be greater at certain salinities without noticeable changes in total dry weight and protein content^10,15^ thus specific growth and division rates (Fig. 2b) may not necessarily reflect final biomass yields. The observed non-linear growth patterns (Fig. 2a,b) in response to salinity could point to adaptive flexibility adopted by mangrove-dwelling species like UMT-M1^11^ and could have been more beneficial for mangrove habitat dynamics. The results thus affirm that UMT-M1 has euryhaline properties and is capable of easily adapting to different salinities.

The photosynthetic apparatus is the microalgae’s first line of defence against an array of stress conditions. Chlorophyll content is therefore associated with growth kinetics reflective of internal conditions^16^. Reduction in chlorophyll content had always been a classic sign of oxidative stress in microalgae cultures undergoing salinity stress. The results indicated that although chlorophyll content in all salinities, with the exception of 15 ppt, were significantly lower than the control, chlorophyll content was not drastically reduced nor was growth disrupted (Fig. 2a,b). Conservation of chlorophyll content had been suggested to act as a compensation mechanism towards the impending effects of fluctuating salinity levels^17^; in other words, osmoregulation. Since photosynthesis biochemically fixes carbon into energy reserves, this could also imply that relevant biochemical carbon fixation activities towards product biosynthesis (i.e. carbon partitioning) was continuous into stationary growth phase. This, therefore explains why chlorophyll content (Fig. 2c) was observed corresponding more closely to the specific growth rate, division rate and oil content (Fig. 2b) rather than biomass accumulation as reported in *Neochloris oleoabundans*^18,19^. Osmoregulation is achieved by means of increasing photon flux density towards supporting the energy demands of setting up an efficient protective mechanism against toxic salt ions^17^ thus allowing growth to proceed at different salinity levels.

### The effect of different salinity levels on total oil content

It had been observed that lower levels of salinity stress ranging from 0.06 to 0.4 M NaCl (or ~ 3.5 to 23 ppt) increased lipid content of freshwater *C. vulgaris* but was coupled with a loss in biomass productivity during high lipid accumulation^12,20^. In a separate study, lipid content of freshwater *C. vulgaris* was successfully increased from 16.7 to 23%) at 0.02 M NaCl (~ 1.2 ppt) with uncompromised growth, albeit, under mixotrophic mode^21^. The current results indicated that *C. vulgaris* UMT-M1 was able to grow well in salinities higher than what is mostly reported in the literature and managed to accumulate > 35% total oil content (Fig. 2d). In oleaginous microalgae, lipid accumulation, normally TAG, is triggered under stressful conditions that compromise cell growth with reductions in the photosynthetic apparatus^22–24^. Recently, salinity-stress triggered lipid accumulation had been thought to be a defence mechanism against osmosis-induced cell volume shrinkage^16,25^. By extension of this idea, it was observed that *C. vulgaris* UMT-M1 accumulated high total lipid (> 35%) across the tested salinities. Coincidentally, it was also observed that brackish-water microalgae such as *Scenedesmus obliquus* and *Phaeodactylum tricornutum* could, on average, accumulate higher total lipid content than marine-isolated microalgae like *Chaetoceros muelleri* at their stationary phase^26^. Since growth parameters were maintained (Fig. 2a–c), this exemplifies an adaptive process, where *C. vulgaris* UMT-M1 dynamically incorporates itself with high intracellular lipid content which would enable *C. vulgaris* UMT-M1 to respond instantaneously amidst fluctuating salinity in mangrove areas.

Collectively, good biomass productivity (Fig. 2a), high chlorophyll content (Fig. 2c) and high total oil content (Fig. 2d) were observed at the intermediate salinity level of 15 ppt. It is postulated that different salinity levels provide different levels of salt ions in culture medium which could have affected metabolic flux in *C. vulgaris* UMT-M1. This could explain why, high chlorophyll content had coincided with highest total oil content at the intermediate salinity level of 15 ppt, but not in 30 ppt even though both salinity levels have maintained similar chlorophyll content.. Moreover, emerging studies targeting alternative signal transduction pathway proteins like ATP-competitive target of rapamycin (TOR) inhibitors and *ZnCys* gene receptors show that lipid production in microalgae can be genetically modified in order to successfully trigger oil accumulation without compromising biomass yield and photosynthesis^27–29^. Trailing from the above studies, it is thus suggested that there may be inherent ‘trigger-pathways’ in mangrove-dwelling microalgae such as UMT-M1 that enable it to achieve both high oil content and uninterrupted growth at certain concentrations of external salinity fluctuation. In *C. vulgaris* UMT-M1, the lipid ‘trigger’ was likely to be activated at 15 ppt salinity level. Mangrove-isolated species could warrant further studies as this characteristic may hold the key to overcoming the high yield—low biomass conundrum experienced in microalgae applications.

### Effect of different salinity levels on fatty acid composition of *C. vulgaris* UMT-M1

Total SFA, MUFA and PUFA contents were fairly consistent across the different salinity levels but changes were detected for several individual fatty acids (Fig. 3). Highest salinity (30 ppt) promotes the synthesis of C16:0 SFA, while the lowest salinity (5 ppt) promotes C18:0 production. Higher pools of C16:0 (46.4%) at 30 ppt could possibly point towards remnants of membrane structure reinforcement^1^. Although unsaturated fatty acids of C18 varieties, i.e. C18:1 and C18:2 exhibited a fluctuating accumulation trend, the C18:3n3 production continued to trend higher along the increasing salinity levels (Fig. 3). Studies carried out on freshwater *Botryococcus braunii* demonstrated a drastic decrease in C18:0 content with increasing salinity^30^. Moreover, omega-3 PUFA was observed to be present in higher levels in brackish and freshwater phytoplankton^31^. Conversely, it has been reported that higher salinity levels led to increased PUFA content in halophilic microalgae like *Dunaliella salina*, where the accumulated PUFA was used in support of cellular membranes^32,33^. These points toward a regulatory role played by C18 varieties in *C. vulgaris* UMT-M1 for mitigating fluctuations from high to low salinities and vice versa.

### Biomass and oil production of *C. vulgaris* UMT-M1 at different growth phases under 15 ppt salinity measurement

The 15 ppt salinity level was further studied on the basis that it induced highest oil accumulation in *C. vulgaris* UMT-M1 cultures (Fig. 2d). The gradual increase of total oil content which span from early exponential (day 2) to mid-stationary (day 12) may point towards general favour of carbon partitioning towards lipid metabolism in *C. vulgaris* UMT-M1. However, the subsequent decrease at the late stationary growth phase (day 15) contradicted biomass production which was the highest at day 15 (Fig. 4a). This suggests that accumulation of other compounds may have taken place at the late stationary phase. It has been reported that starch accumulation was triggered in the euryhaline microalga *Tetraselmis subcordiformis* when placed under lowered salinity conditions coupled with nitrogen limitation^34^. The current results signalled that the cultivation of *C. vulgaris* UMT-M1 under 15 ppt triggered lipid accumulation maximally starting from early exponential growth phase, and way up to mid-stationary phase before switching off lipid production in favour of other metabolic pathways in the late stationary phase (Fig. 4b). Previously, the switch from lipid to starch had been reported to be characteristic of brackish-water microalgae^35^.

### Influence of different growth phases on fatty acid composition of *C. vulgaris* UMT-M1 cultivated under 15 ppt salinity measurement

TAGs act as repositories of stable inert fatty acid building blocks that the cell can instantly draw from or store for future usage^34,35^. Distinct properties of individual fatty acids are conferred through the length and degree of saturation of the fatty acyl chains^1,4^. Hence, the diverse fatty acid types incorporated into TAG storage is reflective of the cell’s needs and lipid metabolism as cultures switched from 30 to 15 ppt conditions. This was observed to progress successively as the microalgae culture moved through its different growth stages. Early stage cultures (day 2–day 5) were characterized by the presence of more diverse fatty acid types, particularly the short-chain (SC) SFAs (C6:0 and C12:0), medium-chain (MC) SFAs (C14:0, C15:0) and long-chain (LC) SFA (C20:0), besides the commonly detected major fatty acids such as C16:0, C18:0, C18:1, C18:2 and C18:3n3 (Fig. 4d, Supplementary Fig. S2). Deposition of SC-/MC-SFAs in TAG at early exponential phase, particularly day 2, may indicate operations by the cell as it tries to quickly detect and counter the change in salinity level. While limited studies in microalgae recognize the involvement of SC-/MC-SFAs response to stress^36^, studies on mammals have found that administering SC-/MC-SFAs did help alleviate stress in mice^37^. Shorter hydrocarbon chains have also been acknowledged to possess characteristics such as low viscosity, low freezing points, low surface tensions with good oxidation stability and high antioxidant abilities^1,36^ and is thus suitable for maintaining overall lipid order. Day 2 TAG was also earmarked with very high amounts of C18:3n3 PUFA and C20:0 SFA with minute amounts of C20:3n6 PUFA (Fig. 4d, Supplementary Fig. S2). This disproportion (Supplementary Fig. S3b) is caused by a preference in lipid metabolism for PUFAs and SFAs over C18 MUFA (in particular C18:1) in early- and mid-exponential phase (day 2–5) cultures. Increment in PUFA, particularly the C18:3n3, in TAG not only points to an increase in *ω* − 3* FAD* gene activity^38^ but could also point to its prerogative of a maintenance role, particularly in the preservation of chloroplast structures and conveying more fluidity to membranes^39^. The C20:3n6 PUFA (Supplementary Fig. S2) on the other hand is a known a precursor to C20:4n6, a PUFA which has been associated with an ecological role of signalling growth responses^1^. Notably, PUFA products could be incorporated as key intermediates in cell signalling pathways^40^. In higher plants, PUFA production has been attributed to a role in sensing changes in the environment^41^ and can possibly explain the elevated amount of PUFA in day 2 cultures. Elevated PUFA and SFAs in the TAG could have provided a reservoir of appropriate building blocks for further use as membrane reinforcements and stress regulators to aid *C. vulgaris* UMT-M1 in maintaining proper cell growth and overcoming the initial lag phase, caused by direct salinity change in the outer environment.

More fatty acid types disappear from TAG storage as culture phase progressed. When cultures moved towards the stationary phase, generally lesser fatty acid types (in particular only five major fatty acids) were partitioned into TAG storage (Fig. 5, Supplementary Fig. S3b). The C18:0 types, predominantly C18:1 and C18:2 were seen to increase towards mid- and late-stationary phase. It is postulated that pools of C18:1/C18:2 accumulate so that it can be readily converted into C18:3n3 via *ω* − 3*/ω* − 6* FAD* enzymes during the next wave of salinity level change or exponential growth encountered by the cells thus providing a pool of ready-to-use C18:3n3 which was a very important growth and stress modulator in *C. vulgaris* UMT-M1. The significant role of C18:3n3 PUFA during active cell growth was clearly reflected by the steep and continuous declining trend of its accumulation to a minimum amount towards mid- and late-stationary growth phases (Fig. 5). Proportions of SFAs, particularly the predominant C16:0, was constantly maintained at a high level across all growth phases and can be attributed to energy conservation in the cell. TAG high in saturated fatty acid gives the microalgae a competitive evolutionary advantage particularly because it is less energy taxing to synthesize cell intermediates from SFAs plus higher amounts of energy can be released upon SFA oxidation^1,23^.

Overall, salinity fluctuation (as cultures moved from 30 to 15 ppt) was observed to automatically evoke adaptative capacities via a diverse repertoire of saturated fatty acid types as *C.vulgaris* UMT-M1 battles through the initial period of fastidious adjustments and active cell growth. Elevated PUFA levels, especially C18:3n3 in *C. vulgaris* UMT-M1 could have played an important functional role in maintaining cell conditions or in eliciting adaptation. The fatty acid profile gradually becomes relatively distinct with a select few fatty acid types (predominantly C16:0, C18:0, C18:1, C18:2, C18:3n3, C20:0) at stationary phase (Fig. 4d; Supplementary Fig S2) showing that internal cellular responses are highly regulated and controlled once the changes in salinity have been detected and tackled by the cell.

## Materials and method

### Culture condition and salinity treatments

*C. vulgaris* UMT-M1^11^ was obtained from the microalgae culture collection of Universiti Malaysia Terengganu. Inoculum was initiated from a single colony from purified stock agar plate and established in Guillard’s F2 media^42^ prepared with 2.5 L of 30 ppt artificial seawater (Instant Ocean Salt, Aquarium Systems). Cells were maintained in 3 L Erlenmeyer flasks under sterile axenic conditions (temperature of 24 ± 2 °C; 24 h cool daylight-emitting diode (LED) lighting at ~ 140 μmol m^−2^ S^−1^) and constant aeration with filtered air (0.22 µm filter membrane, Sartorius AG, Germany).

Cell density readings were monitored daily in triplicates. Cell concentration was first obtained by aliquoting 1 mL of cell sample into Liquid Particle Counter-SLS-2000 (Particle Measuring System, USA) while absorbance wavelength measurements was recorded at OD_681_ using micro plate reader VarioskanTM LUX (Thermo Fisher Scientific, USA). Cell numbers were correlated with OD_681_ values to obtain cell calibration curve.

Salinity treatments were carried out in two stages with all final salinity values maintained at initial salinity values. In the first stage, various salinity levels were screened to determine the salinity level that rendered highest total oil production. In this experiment, the F2 medium was prepared using artificial sea water (Instant Ocean, Aquarium Systems) at salinity levels of 5, 10, 15, 20 and 30 ppt. The salinity level of 30 ppt was used as control as *C. vulgaris* UMT-M1 was originally maintained and cultured under this salinity condition. The inoculum size was standardized at 1 × 10^7^ cells mL^−1^ (from 30 ppt cultures) and the cells were inoculated into lower salinities media for cultivation. The experiment was carried out in three biological replicates. Growth of cultures was monitored daily until day 12 where the cultures achieved stationary growth phase and the cells were harvested for the analysis of cell growth, biomass productivity, chlorophyll content, total oil content and fatty acid composition.

In the second stage, the salinity level that gave the highest oil production was used and further investigated for the effect of different growth stages on oil and fatty acid productions. All culture conditions were as outlined above except that assess points were fixed at early exponential (day 2), mid-exponential (day 5), early stationary (day 8), mid stationary (day 12) and late stationary (day 16) stages. Three biological replicates of the cultures were randomly selected and harvested for analysis of cell growth, biomass productivity, total oil content and fatty acid composition.

### Biomass production, biomass productivity and specific growth rate

The following measurements were carried out in triplicates across all salinity treatments. Freshly harvested cells (centrifugation at 7000 rpm; 5 min) was washed with ammonium bicarbonate (NH_4_HCO_3_) followed by a round of distilled water flush. The cells were then dried overnight in an oven at 60 °C. Total biomass dry weight (DW) was calculated as reported^6^.

Volumetric biomass productivity (g L^−1^ day^−1^) was calculated using the following equation^43^:

$$ {\text{g}}\,{\text{L}}^{ - 1} \,{\text{day}}^{ - 1} = \frac{{({\mathcal{X}}_{1} - {\mathcal{X}}_{2} )}}{{t_{1} - t_{2} }} $$

where ${\mathcal{X}}_{1}$ and ${\mathcal{X}}_{2}$ are biomass dry weight collected at $t_{1}$ (inoculum; start of cultivation) and $t_{2}$ (end of cultivation).

Specific growth rate (*µ*) was measured according to the following equation^44^:

$$ \mu = \frac{{ln \left( {N_{2} - N_{1} } \right)}}{{t_{2} - t_{1} }} $$

where $N_{2}$ and $N_{1}$ are cell density readings collected at $t_{1}$ (start of exponential phase—day 2) and $t_{2}$ (end of exponential phase—day 8).

Division rate of cells ($k$) was measured according to the equation^44^:

$$ k = \frac{\mu }{\ln 2} $$

### Chlorophyll content

The chlorophyll was extracted and measured according to modified method by Ritchie^45^. Fresh pelleted cells (1 mL from culture) was re-suspended in 95% (v/v) methanol (Merck, ACS grade). The mixture was then heated to 60 °C for 5 min with a heating block in order to facilitate extraction of chlorophyll pigments. The cells were left to incubate for 24 h at 4 °C in the dark. After 24 h, cell debris was removed via centrifugation and supernatant was quantified against a blank using micro plate reader Varioskan™ LUX (Thermo Fisher Scientific, USA). Chlorophyll content (µg mL^−1^) was then quantified according to the modified equation of Ritchie^45^:

$$ \begin{aligned} {\text{Chlorophyll}}\,{\text{a}}\,\left( {{\text{chl}}\,{\text{a}}} \right):\left( {\upmu {\text{g/mL}}} \right) & = 16.72\,A_{665.2} - 9.16\,A_{652.4} \\ {\text{Chlorophyll}}\,{\text{b}}\,\left( {{\text{chl}}\,{\text{b}}} \right):\left( {\upmu {\text{g/mL}}} \right) & = 34.09\,A_{652.4} - 15.28\,A_{665.2} \\ \end{aligned} $$

### Total oil content and fatty acid composition analysis

The total oil was extracted and determined following the protocol as previously described with minor modification^6^. Fraction of dried biomass (0.3 g) was crushed into fine powder using a mortar and subsequently immersed in 10 mL of concentrated HCl. (37%) The mixture was gently vortexed for 2 min and double-boiled at 100 °C for 20 min to induce cell disruption. Upon completion, the test tubes were allowed to cool to room temperature. Subsequently, the lipid in the solution was extracted once with 12.5 mL hexane and twice with 7.5 mL hexane. The mixture was shaken vigorously and the lipid layer was collected in an empty flat bottom flask. The combined hexane extract layers were then evaporated via rotary evaporator (Rotavapor R-300, Buchi, Switzerland) for 30 min.

The triacylglyceride (TAG) portion of total oil extract was converted into fatty acid methyl esters (FAME) according to method previously described^6^. A total volume of 5 mL 0.5 N NaOH (in methanol) and some boiling chips were gently mixed with 50 mg of oil in a flat bottom flask. The flask was then attached to a Lienberg Condenser and its contents boiled with a heating mantle for 10 min followed by addition of 5 mL of boron-trifluoride (in 20% methanol). The mixture was boiled for 2 min followed by addition of 2 mL of *n-*heptane and boiled for another 1 min afterwards. The flask was then removed from heating mantle and left to cool. After that, 15 mL of saturated sodium chloride was added into the mixture before transferring the mixture into a test tube where its contents were left to stand momentarily. The upper layer supernatant (FAME extract) was then collected into 1.5 mL vials.

Esterified microalgae oil was further analyzed using Shimadzu GC-2010 *plus* gas chromatography with flame-ionization detection (Shimadzu, Japan) on a HP-88 capillary column (Agilent Technologies, USA). Chromatography was performed using helium as carrier gas with a constant flow rate of 2 mL min^−1^. Injection parameters: split injection was performed with ratio 1:50 at 250 °C. The oven temperatures applied were 175 °C for 10 min and a 15 min hold when temperatures were increased to 250 °C at 3 °C min^−1^. Annotation of chromatogram peaks was performed comparing peak and retention time of external reference to a standard (Supelco 37 Component FAME Mix; Sigma Aldrich, USA).

### Statistical analysis

All data were analysed using one-way ANOVA and followed up with Fisher’s LSD multiple comparison test with the confidence level set at *p* < 0.05. End-point of the stationary phase of growth was determined by three consecutive points that are not significantly different from each other. Analysis was carried out using SPSS software (IBM SPSS Statistics 26.0).

Multivariate analysis was applied to analyse FAME output from GC-FID. For the first stage of experiment, comparisons of FAME data between different salinities were analysed using Principal Component Analysis (PCA). In the second stage of experiment, variation of FAME within different culture days was analysed using Partial Least Squares Discriminant Analysis (PLS-DA). All sample values were linear-logged and Pareto-scaled. Analysis was carried out in SIMCA (16.0 Umetrics; Sartorius) software.

## Conclusion

A major remaining concern from many stress-mediated strategies is the high-yield-low-biomass conundrum. Hence, this paper is a promising addition which demonstrated that certain salinity levels may trigger TAG accumulation in mangrove-isolated *C. vulgaris* UMT-M1 with uncompromised biomass productivity and healthy chlorophyll levels without genetic enhancement. Moreover, the dynamic changes observed in the fatty acid profile at different growth stages show that fatty acid of differing acyl chain lengths play significantly important roles in adaptation to salinity fluctuation and in growth. It does appear that the roles of different fatty acyl chain lengths still remain relatively unknown in microalgae. At this stage moving forward, projecting the biochemical observations in this study onto currently known molecular pathways will allow us to gain insights and aid in pinpointing the possible genomic, transcriptomic proteins or signalling pathways that allow for triggered lipid accumulation.

## Supplementary Information

Supplementary Information.
