# Supplementary material for: Lipid accumulation patterns and role of different fatty acid types towards mitigating salinity fluctuations in Chlorella vulgaris
Source: Sci Rep. 2021 Jan 11;11:438. doi: 10.1038/s41598-020-79950-3 (PMC7801682; doi:10.1038/s41598-020-79950-3)
Supplement: Supplementary file 1 — Supplementary Information. [file 41598_2020_79950_MOESM1_ESM.docx]

**SUPPLEMENTARY MATERIALS**

**Lipid accumulation patterns and role of different fatty acid types towards mitigating salinity fluctuations in *Chlorella vulgaris***

**Author names and affiliations**

Kit Yinn Teh^2,3^, Saw Hong Loh^1,2^, Ahmad Aziz^1,2^, Kazutaka Takahashi^4^, Mohd Effendy Abd Wahid^2,3^, Thye San Cha^1,2,*^

^1^Faculty of Science and Marine Environment, Universiti Malaysia Terengganu, 21030 Terengganu, Malaysia

^2^Satreps-Cosmos Laboratory, Central Laboratory Complex, Universiti Malaysia Terengganu, 21030 Terengganu, Malaysia

^3^Institute of Marine Biotechnology, Universiti Malaysia Terengganu, 21030 Terengganu, Malaysia

^4^Department of Aquatic Bioscience, Graduate School of Agricultural and Life Sciences,
The University of Tokyo, 1-1-1, Yayoi, Bunkyo-ku, Tokyo, 113-8657, Japan

*** Corresponding author: Cha TS.**

Tel: +609-6683394 Fax: +609-6683193 E-mail: cha_ts@umt.edu.my


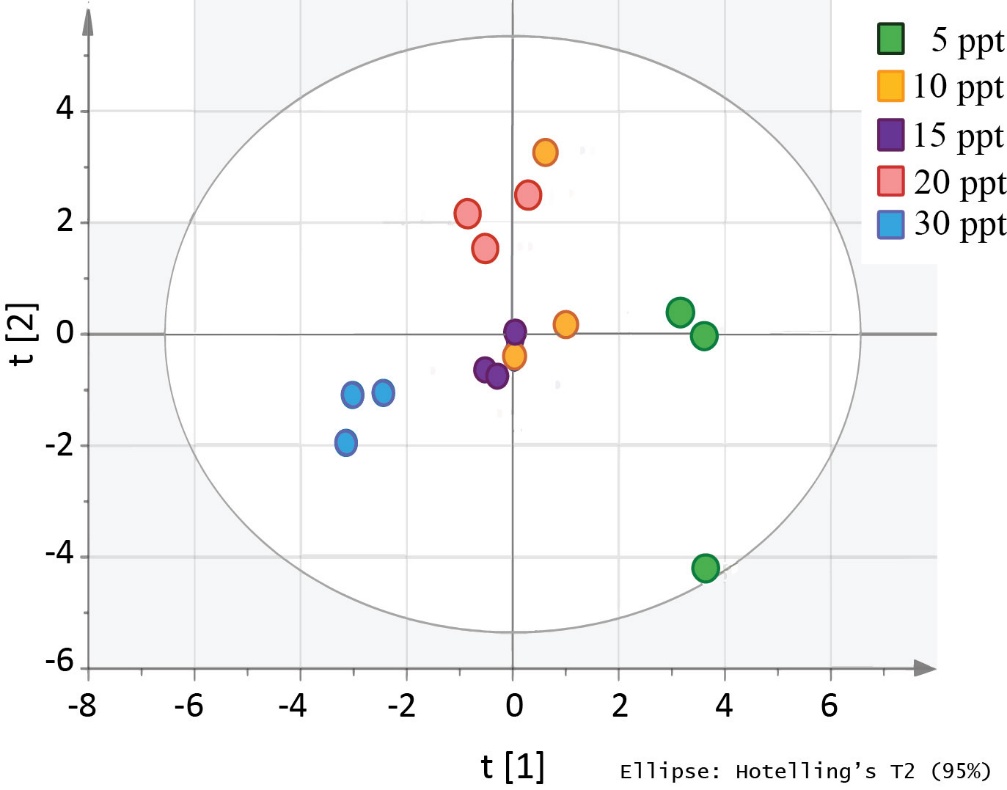


**Supplementary Fig. S1:** PCA score plot (SIMCA) of the fatty acid profiles obtained from GC-FID analysis of different salinities (5, 10, 15, 20, 30 ppt); *n* = 15. Each dot of the same colour represents the overall FAME profile of a single replicate. The first component (t[1]) explains 40.4% of the variance while the second component (t[2]) explains 27% of the variance. Eclipse on plot represents 95% confidence level. R^2^X = 0.894, Q^2^= 0.201.


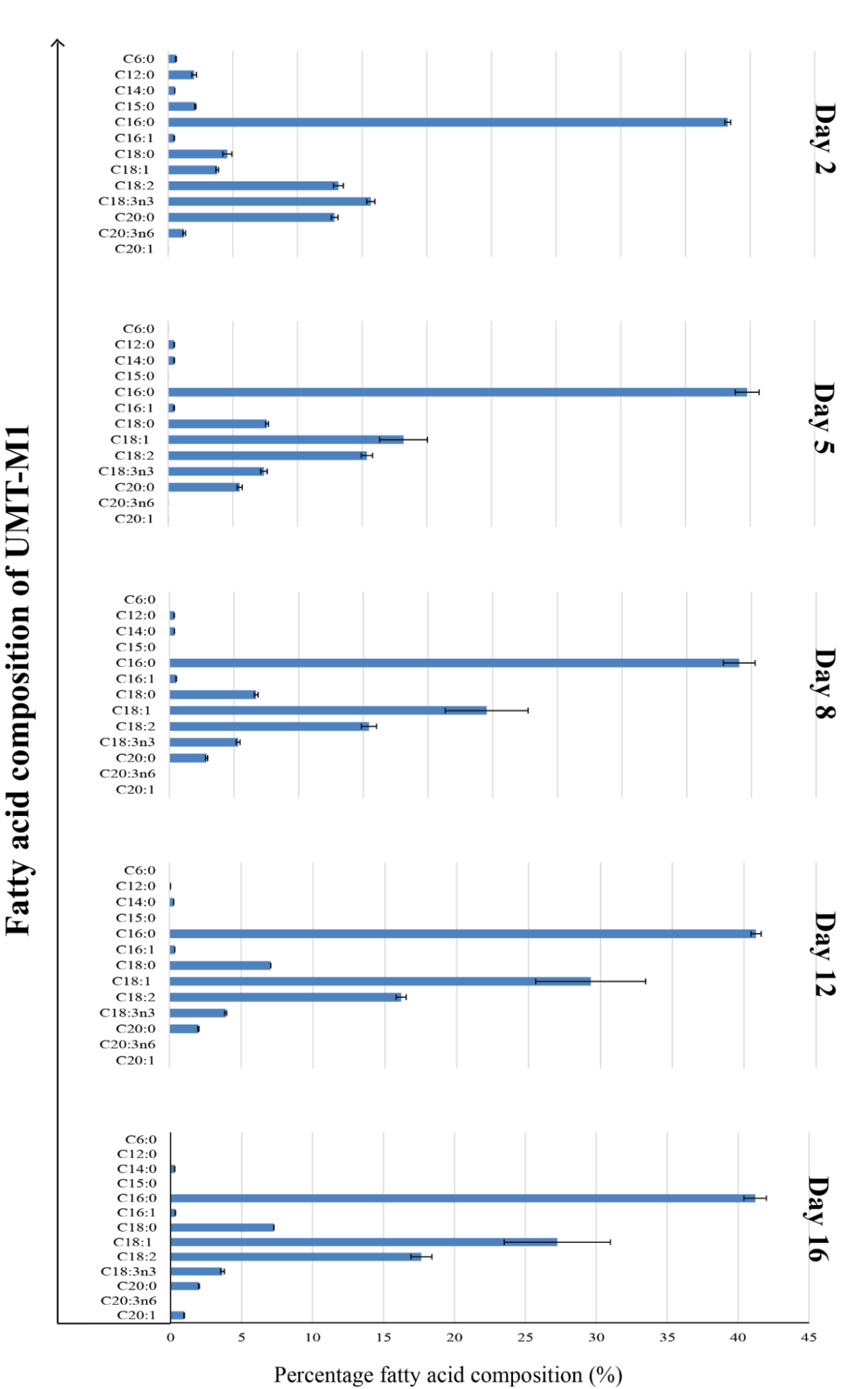
 **Supplementary Fig. S2:**Breakdown of fatty acid component over day 2, 5, 8, 12 and 16 of UMT-M1 under 15 ppt culture conditions.


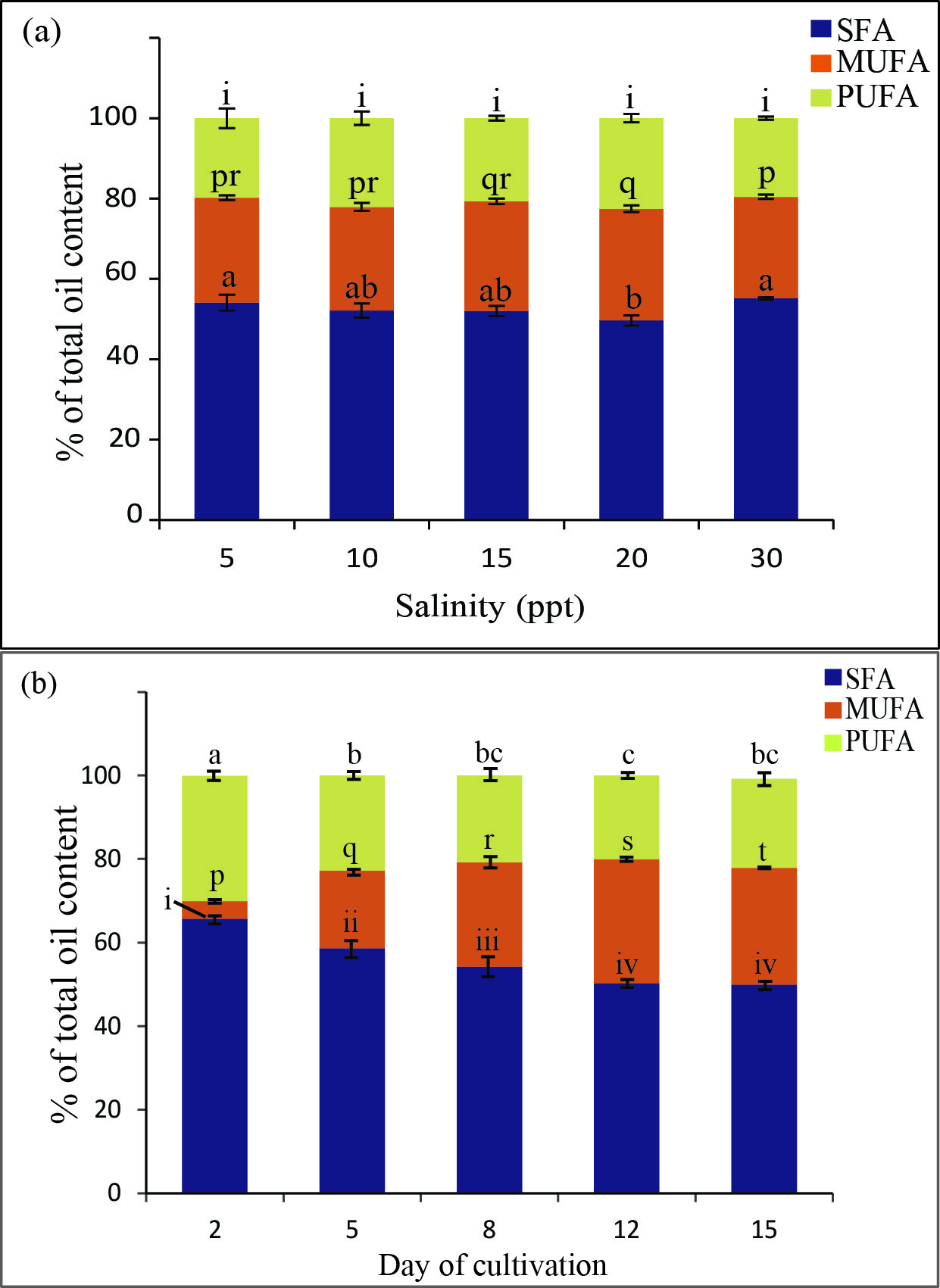


**Supplementary Fig. S3:** Distribution of changes in overall fatty acid groups cultured **(a)** under different salinity levels at stationary phase and **(b)** during different growth phases (measured from day 2 – day 16) under the salinity level of 15 ppt in *C.vulgaris* UMT-M1. All values represent means ± SD of three biological replicates. Different series of letters are significantly different according to Fisher’s LSD test (SPSS software) (*p* < 0.05). SFA: Saturated fatty acid; MUFA: Monounsaturated fatty acid; PUFA: Polyunsaturated fatty acid

**Table S1 (Supplementary)** Cell density of *C. vulgaris* UMT-M1 cultured under different salinities at stationary phase (day 12). Data presented are of means ± SD of three biological replicates. Different letters observed vertically indicate significant differences according to Fisher’s LSD at (p < 0.05).

| Salinity (ppt ) | Cell density  (×10^8^ cells mL^-1^) |
| --- | --- |
| 5 | 2.4 ± 0.021 ^bc^ |
| 10 | 2.1 ± (0.043) ^d^ |
| 15 | 2.6 ± (0.08) ^a^ |
| 20 | 2.5 ± (0.028) ^ab^ |
| 30 | 2.3 ± (0.01) ^c^ |
